# Longitudinal 3D‐DCE MRI Assessment of Placental Perfusion Under Reduced Uterine Perfusion Pressure in Pregnant Rats

**DOI:** 10.1002/nbm.70251

**Published:** 2026-02-22

**Authors:** Fatimah M. Al Darwish, Caren M. van Kammen, Fieke Terstappen, Lindy K. Alles, Raymond M. Schiffelers, A. Titia Lely, Gustav J. Strijkers, Bram F. Coolen

**Affiliations:** ^1^ Department of Biomedical Engineering and Physics Amsterdam UMC, University of Amsterdam Amsterdam the Netherlands; ^2^ Amsterdam Cardiovascular Sciences Amsterdam the Netherlands; ^3^ Department of CDL Research University Medical Center Utrecht, Utrecht University Utrecht the Netherlands; ^4^ Department of Obstetrics, Wilhelmina Children's Hospital University Medical Center Utrecht, Utrecht University Utrecht the Netherlands; ^5^ Department of Neonatology, Wilhelmina Children's Hospital University Medical Center Utrecht, Utrecht University Utrecht the Netherlands

**Keywords:** 3D‐DCE MRI, placental imaging, placental perfusion, RUPP

## Abstract

Placental insufficiency is a major cause of preeclampsia and fetal growth restriction. The reduced uterine perfusion pressure (RUPP) rat model reproduces cardinal features of these disorders, but longitudinal changes in placental perfusion after RUPP are poorly documented. This study aimed to assess the acute and late effects of RUPP on placental perfusion between gestational day (GD) 14 and GD18 using 3D dynamic contrast‐enhanced magnetic resonance imaging (3D‐DCE MRI). Pregnant Sprague Dawley rats (*n* = 31) were randomized to RUPP or normal pregnant (NP) groups on GD14. 3D‐DCE MRI scans were performed on GD14, GD15, and GD16 or GD18. Perfusion parameters, including time to peak (TTP), maximum enhancement (ME), wash‐out percentage (WO%), initial area under the curve (iAUC), and total area under the curve (AUC), were derived from fitted signal–time curves. In addition, exploratory voxel‐wise histogram analysis was performed to assess intralitter variability. At GD14, RUPP placentas showed delayed TTP (*p* < 0.0001), reduced ME (*p* = 0.03), reduced WO% (*p* < 0.0001), and elevated iAUC (*p* = 0.02). Differences persisted for TTP (*p* = 0.02) and WO% (*p* = 0.01) at GD15 but resolved by GD16–GD18. Voxel‐wise analysis revealed intralitter heterogeneity, with 20% of analyzed RUPP placentas deviating from the NP template. These findings indicate that RUPP induces an acute but transient impairment of placental perfusion, with recovery by late gestation. GD14–GD15 emerge as a critical window for detecting acute perfusion impairment and evaluating interventions targeting acute injury.

## Graphical abstract

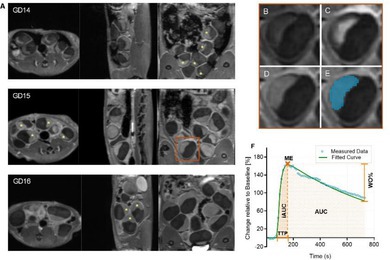

**Abbreviations**
AUC: area under the curve
FGR: fetal growth restriction
GD: gestational day
iAUC: initial area under the curve
JSD: Jensen–Shannon distance
ME: maximum enhancement
NP: normal pregnant
PE: preeclampsia
RUPP: reduced uterine perfusion pressure
TTP: time to peak
WO%: wash‐out percentage

## 1 Introduction

The placenta is a dynamic organ that facilitates nutrient and oxygen exchange between maternal and fetal circulations and provides essential regulatory support for fetal development. Placental insufficiency, associated with impaired blood flow, underlies major pregnancy complications such as preeclampsia (PE) and fetal growth restriction (FGR) that are associated with preterm birth and significant perinatal morbidity and mortality [1]. Currently, no effective treatments for placental insufficiency exist, and clinical assessment relies largely on indirect Doppler ultrasound indices (e.g., uterine and umbilical artery pulsatility and absent/reversed end‐diastolic flow) rather than direct measures of placental function [2]. These unmet needs require improved monitoring strategies alongside the development and testing of new therapeutic interventions. As direct investigation of pregnancy disorders in humans is limited by ethical constraints and potential risks to mother and fetus, animal models are essential for advancing our understanding of placental function and for preclinical evaluation of therapeutic interventions.

Several animal models have been established to study PE, including approaches based on artery ligation, nitric oxide inhibition, renin‐angiotensin system modulation, anti‐angiogenic factor exposure, immunological manipulation, and transgenic strategies [3]. FGR has been induced through dietary restriction, heat exposure, artery ligation, hypoxia, embolization, or glucocorticoid administration [3]. Among these models, the reduced uterine perfusion pressure (RUPP) rat model is widely used because it replicates many hallmarks of human PE, including hypertension, proteinuria, endothelial dysfunction, and FGR [4]. The RUPP model is a mechanical model that involves partial clipping of the abdominal aorta to reduce uterine perfusion with additional clipping of the ovarian arteries to limit compensatory flow [4].

The RUPP model has been used to study placental metabolism [5], oxygenation [6], oxidative stress [7], inflammation [8], and potential therapeutic interventions, including tempol [9], sildenafil [9, 10], and vitamin D [11]. However, functional assessment of placental perfusion in this model is limited. In particular, little is known about how placental perfusion changes throughout gestation following RUPP surgery. Such information is critical to understand pathophysiological adaptations and define windows for therapeutic interventions.

Dynamic contrast‐enhanced magnetic resonance imaging (DCE‐MRI) enables noninvasive assessment of tissue perfusion using gadolinium‐based contrast agents. Although its clinical use in pregnancy is restricted due to potential fetal risks, DCE‐MRI is well suited for preclinical studies. We have previously demonstrated that 3D‐DCE MRI can assess perfusion in all placentas in utero, highlighting its utility for longitudinal placental studies [12]. In that study, measurements were performed at gestational day (GD) 19 only. Notably, we observed no difference in placental perfusion between RUPP and control pregnancies. However, whether this restoration of placental perfusion occurs progressively over time remains unknown.

The objective of this study was therefore to investigate the acute and late gestational effects of RUPP surgery on placental perfusion from GD14 to GD18 using longitudinal DCE‐MRI in pregnant rats. We hypothesize that placental perfusion would be impaired immediately after RUPP surgery but would exhibit partial to full compensatory adaptations at later gestational stages.

## 2 Material and Methods

All experimental procedures were approved by Amsterdam University Medical Centers Animal Ethics Committee and conducted and reported in accordance with the ARRIVE guidelines and European Union guidelines for the welfare of laboratory animals (Directive 2010/63/EU).

### 2.1 Animals

Eleven‐ to 13‐week‐old timed pregnant Sprague Dawley rats (*n* = 31) were supplied by Envigo (Envigo RMS B.V., Horst, the Netherlands) at GD8. Animals were housed in pairs in conventional cages under a regular 12‐h light/dark cycle, at a constant room temperature (22°C) and humidity (50%), with free access to food and water. A 5‐day acclimation period preceded the experiment. To minimize confounders, cage positions and the daily order of imaging were randomized.

### 2.2 RUPP Procedure

Pregnant dams were randomly assigned by simple randomization to either the RUPP or normal pregnant (NP) group. The RUPP procedure was performed as previously described [12]. Briefly, animals were put under isoflurane anesthesia (3% induction, 2%–2.5% maintenance), after which a midline laparotomy allowed exposure of the uterine horns. A silver clip (AG000450, Goodfellow Cambridge, Pittsburgh, PA, USA) with dimensions of 15‐mm length, 1.5‐mm width, and 0.203‐mm inner diameter was placed around the abdominal aorta above the bifurcation of the iliac arteries, and two additional clips (10‐mm length, 1.5‐mm width, and 0.100‐mm inner diameter) were placed around the right and left ovarian arteries to reduce uterine perfusion. NP animals were anesthetized for a similar duration but did not undergo surgery. NP animals were used as controls to provide a physiological baseline; prior studies report no differences from sham‐operated controls [13]. All animals received carprofen (5 mg/kg, subcutaneous) 30 min before surgery and every 12 h for 24 h postoperatively. After surgery, dams were housed individually until sacrifice. Postoperatively, blinding of both animal care staff and researchers was not feasible because surgical wounds were visible on the animals' bodies. During image analysis, blinding was also compromised by clip‐related artifacts on MRI scans.

### 2.3 MRI Protocol

MRI was performed with a 7.0‐T scanner (MR Solutions Ltd., Guildford, UK) under isoflurane anesthesia (3% for induction, 1%–2% for maintenance) using a quadrature rat‐body volume coil. Body temperature (36°C–37°C) and respiration (40–50 breaths/min) were continuously monitored and kept constant. After short initial scout scans, 3D DCE MRI was performed using a 3D RF‐spoiled gradient‐echo sequence with the following parameters: TR/TE = 8.0/2.0 ms, flip angle = 25°, FOV = 60 × 60 × 60 mm^3^, and matrix = 128 × 96 × 96. No respiratory triggering was performed. Data were acquired for 1 min prior to and 11 min after intravenous administration of Dotarem (0.025 mmol, 0.5 mM Gd; Guerbet) via a tail vein catheter (3 mL/min) using a CHEMYX Inc., Fusion 100 syringe pump. To facilitate retrospective high‐temporal‐resolution reconstructions, data were acquired using a pseudoradial ky/kz trajectory with a golden‐angle increment of 16.95°. Raw data were processed using a publicly available in‐house‐built MATLAB application [14]. The 12‐min:22‐s acquisition was binned into 60 timeframes of 12 s:367 ms/frame. Image reconstruction was performed using the BART toolbox [15] with compressed sensing (BART pics command), employing wavelet regularization (λ = 0.001) and temporal total variation regularization (λ = 0.1).

**FIGURE 1:**
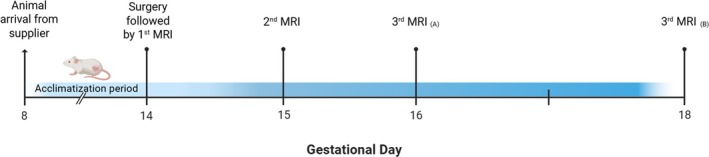
Study timeline. MRI sessions were performed on gestational day (GD) 14 (surgery day), GD15, and either (A) GD16 or (B) GD18 following animal arrival on GD8 and a 5‐day acclimatization period.

Each animal underwent three MRI sessions (Figure 1): on GD14 immediately after surgery, GD15, and either GD16 or GD18. Animals were recovered after each imaging session, except after the final scan, when they were sacrificed.

### 2.4 Conceptus Measurements

Maternal weight was recorded before each imaging session. Dams were euthanized by isoflurane overdose (5%) after the final MRI. Viable and reabsorbed fetuses were counted; placental and fetal weights were measured for each viable fetus. At GD18, one to two fetuses per litter were dissected for measurement of fetal brain and liver weight; this was not feasible at GD16 due to their small size. Following the measurements, all fetuses were sacrificed by decapitation.

### 2.5 Image Analysis

#### 2.5.1 Region of Interest (ROI)–Based DCE Analysis

ROIs on a single sagittal slice per placenta were manually drawn in 3D Slicer (http://www.slicer.org) [16]. Signal–intensity time curves were converted to percent enhancement relative to baseline. These DCE curves were fitted using nonlinear least‐squares optimization (MATLAB, lsqnonlin). Because the DCE profiles varied substantially in shape, three candidate models were tested for each curve: rise with exponential decay, double‐sigmoid, and generalized gamma. Signal‐ and time‐based weighting was applied to emphasize the enhancement peak, and the best model was selected based on the lowest root‐mean‐square error.

**FIGURE 2:**
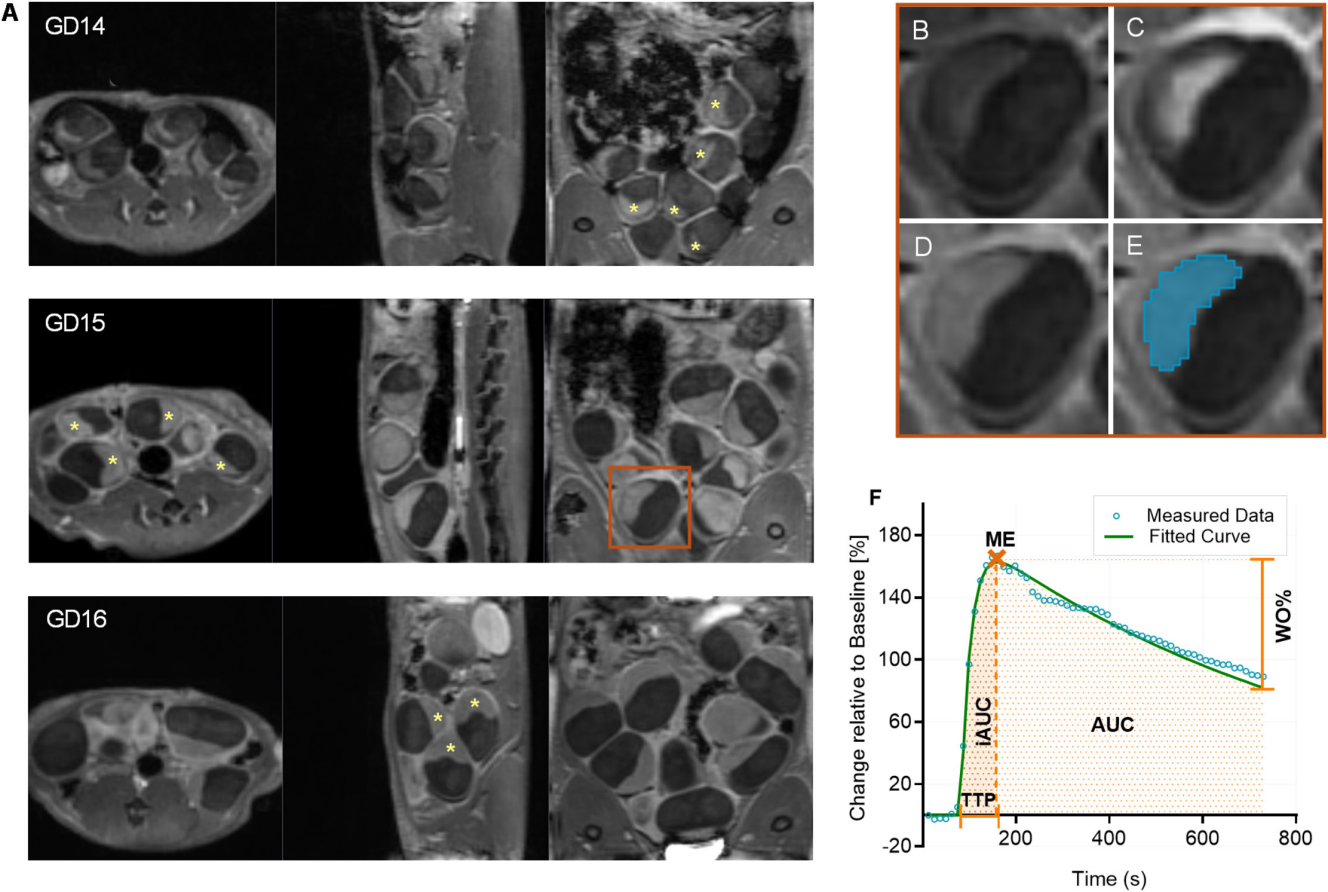
DCE‐MRI analysis workflow. (A) Representative 3D DCE‐MRI datasets of the rat placenta at gestational day (GD) 14, GD15, and GD16, displayed in three orthogonal planes. Placentas are indicated with asterisks. (B–E) Example of a single placenta across different phases: (B) precontrast baseline, (C) maximum enhancement, (D) wash‐out phase, and (E) segmentation. (F) Signal intensity–time curve illustrating measured data (dots) and fitted curve (line). Semiquantitative parameters derived from the curve fit include time to peak (TTP), initial area under the curve to TTP (iAUC), total AUC (AUC), maximum enhancement (ME), and wash‐out percentage (WO%).

Fitted curves were then used for semiquantitative assessment of perfusion parameters: time to peak (TTP), initial area under the curve until the peak (iAUC), total area under the curve (AUC), maximum enhancement (ME), and wash‐out percentage (WO%) as illustrated in Figure 2. Placentas with substantial motion artifacts or associated with nonviable fetuses, identified by clearly reduced size, were excluded.

#### 2.5.2 Voxel‐Wise DCE Analysis

In addition to the ROI‐based analysis, we also performed an exploratory voxel‐wise analysis in four placentas per scan at GD15. To this end, full 3D manual segmentations were performed, selecting two placentas from the upper and two from the lower uterine regions to capture potential spatial variability. Some placentas were excluded when small litter size or motion artifacts prevented segmentation.

From each 3D segmentation, voxel‐wise total AUC values were extracted. As an initial voxel‐wise summary, placenta‐level mean and median voxel‐wise AUC values were computed from the full voxel distributions and compared between NP and RUPP, using linear mixed‐effects models with dam as a random intercept.

To assess distributional differences beyond averaging, voxel‐wise total AUC values were converted into normalized histograms (100 bins, range 0–350,000) to account for differences in placenta size. A reference histogram template was generated by averaging normalized NP histograms. Jensen–Shannon distance (JSD) was calculated to quantify divergence of individual RUPP placentas from the NP template [17], with higher JSD values indicating greater deviation.

### 2.6 Exclusion Criteria

Exclusion criteria were prespecified before data collection to reduce heterogeneity. Animals or datasets were excluded for total fetal resorption and substantial motion artifacts precluding reliable DCE analysis. Criteria were applied across groups and GDs.

### 2.7 Statistical Analysis

Statistical analyses were performed in RStudio (version 4.2.0; R Core Team, Vienna, Austria). Continuous variables were assessed for normality using the Shapiro–Wilk test. For comparing fetal organ weights at GD18, the nonparametric Wilcoxon rank‐sum exact test was used. For measurements that concerned the final time points only (resorption rate and maternal weight gain), two‐way ANOVA was used when ANOVA assumptions were met; otherwise, a nonparametric factorial ANOVA using the aligned rank transform test was applied. Comparisons between RUPP and NP fetal parameters at multiple time points (placental weight, fetal weight, and perfusion parameters) were conducted using a multilevel mixed‐effects model to account for fetal nesting within litters, provided all model assumptions were met. A two‐sided *p*‐value < 0.05 was considered statistically significant. Voxel‐wise histogram analyses were performed in Python (version 3.13; Python Software Foundation, Wilmington, DE, USA). Graphs were created in GraphPad Prism (version 9.1.0; GraphPad Software, San Diego, CA, USA).

## 3 Results

### 3.1 Maternal Outcomes

The percentage of fetal resorption did not differ by group or GD, and there was no group × GD interaction (*p* = 0.87). Maternal weight gain differed between groups, with RUPP dams gaining less weight than NP dams overall (*p* = 0.04) and increasing with gestational age in both groups (*p* < 0.001), but without a group × GD interaction (*p* = 0.25).

### 3.2 Fetal and Placental Morphometric Outcomes

Table 1 presents descriptive statistics for maternal, fetal, and placental outcomes by group and GD. Placental weight increased with gestational age in both NP and RUPP litters (*p* = 0.01), but without a group × GD interaction (*p* = 0.25). Fetal weight increased with gestation in both NP and RUPP litters (*p* < 0.001), without a group × GD interaction (*p* = 0.70). Fetal‐to‐placental weight ratio also increased with gestational age in both groups (*p* < 0.0001), without a group × GD interaction (*p* = 0.28). At GD18, fetal brain and liver weights were similar between NP and RUPP fetuses (*p* = 0.54 and 0.22, respectively), but the brain‐to‐liver weight ratio was higher in RUPP fetuses (*p* = 0.038).

**TABLE 1:** Maternal and fetal outcomes in NP and RUPP groups at GD16 and GD18.

	NP		RUPP	
	GD16(*n* = 7)	GD18(*n* = 8)	GD16(*n* = 8)	GD18(*n* = 7)
Resorptions (%)	8 (12)	4 (15)	27 (38)	15 (42)
Maternal weight gain after GD14 (g)	4 ± 7	20 ± 15	−9 ± 9	16 ± 13
Placental weight (mg)	417 ± 71	519 ± 110	380 ± 59	447 ± 73
Fetal weight (mg)	538 ± 100	1711 ± 431	510 ± 122	1653 ± 383
Fetal brain (mg)	NA	97 ± 16	NA	93 ± 17
Fetal liver (mg)	NA	142 ± 30	NA	122 ± 38
FW:PW (ratio)	1.3 (0.4)	3.1 (0.9)	1.3 (0.4)	3.7 (0.8)
FB:FL (ratio)	NA	0.7 (0.1)	NA	0.8 (0.1)

*Note:* Data are presented as mean ± SD or median (IQR); *n* = number of dams.

Abbreviations: GD, gestational day; NA, not available; NP, normal pregnant; RUPP, reduced uterine perfusion pressure.

### 3.3 DCE‐MRI Perfusion Parameters

Seventeen of a total of 93 DCE datasets needed to be excluded in the analysis, primarily due to the presence of fetal motion during acquisition (GD14: *n* = 4 RUPP and *n* = 2 NP; GD15: *n* = 5 NP; GD16: *n* = 1 RUPP and *n* = 1 NP; and GD18: *n* = 1 RUPP and *n* = 1 NP). Additionally, one RUPP dam was excluded at GD15 and GD18 due to total fetal reabsorption. The final number of animals included in the analysis was as follows: GD14, *n* = 12 RUPP and *n* = 13 NP; GD15, *n* = 15 RUPP and *n* = 10 NP; GD16, *n* = 7 RUPP and *n* = 6 NP; and GD18, *n* = 6 RUPP and *n* = 7 NP.

**FIGURE 3:**
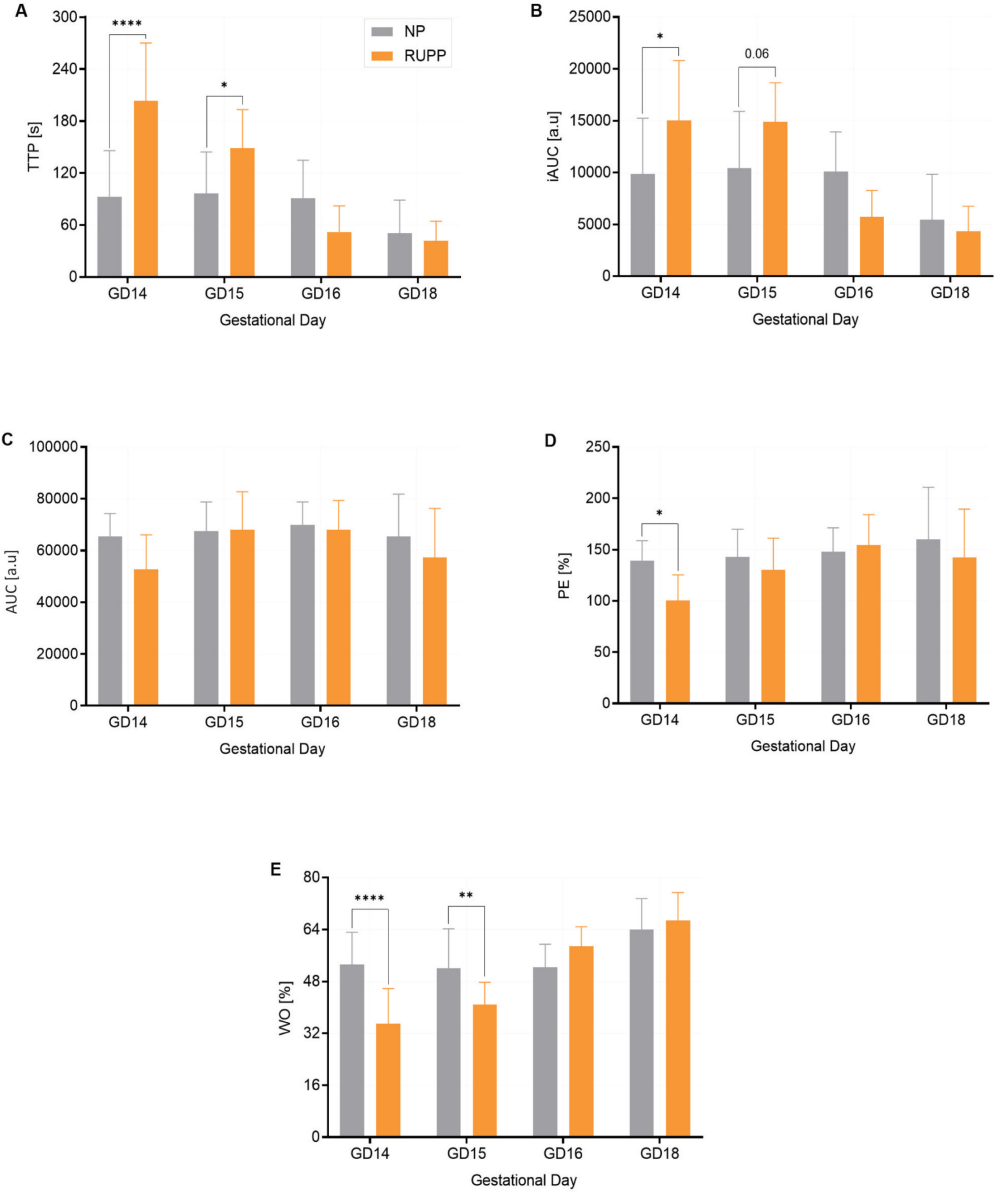
Semiquantitative DCE‐MRI perfusion parameters in RUPP and NP rats. (A) Time to peak (TTP), (B) initial area under the curve to peak (iAUC), (C) total area under the curve (AUC), (D) maximum enhancement (ME), and (E) wash‐out percentage (WO%) measured at GD14, GD15, GD16, and GD18. Data are shown as mean ± SD. Asterisks indicate post hoc pairwise comparisons between RUPP and NP at each gestational day (*p < 0.05, **p < 0.01, ***p < 0.001, ****p < 0.0001).

Longitudinal changes in DCE parameters for RUPP and NP animals are illustrated in Figure 3. For most semiquantitative perfusion parameters (TTP, iAUC, ME, and WO%), a significant group × GD interaction (*p* < 0.001) was found. Specifically, at GD14, RUPP animals exhibited delayed TTP (*p* < 0.0001), higher iAUC (*p* = 0.02), reduced ME (*p* = 0.03), and reduced WO% (*p* < 0.0001) compared to NP. At GD15, differences persisted for TTP (*p* = 0.02) and WO% (*p* = 0.01), with a trend toward higher iAUC values (*p* = 0.06). By GD16 and GD18, no significant differences were observed between groups. Total AUC did not differ between groups across GDs. Video S1 provides representative DCE‐MRI datasets, showing delayed and attenuated enhancement in RUPP at GD14 and normalization by GD16.

### 3.4 Voxel‐Wise Histogram Analysis

Placenta‐level mean and median voxel‐wise AUC did not differ between NP and RUPP at GD15 (mean: estimate −16,186, *p* = 0.229; median: estimate −16,337, *p* = 0.247).

**FIGURE 4:**
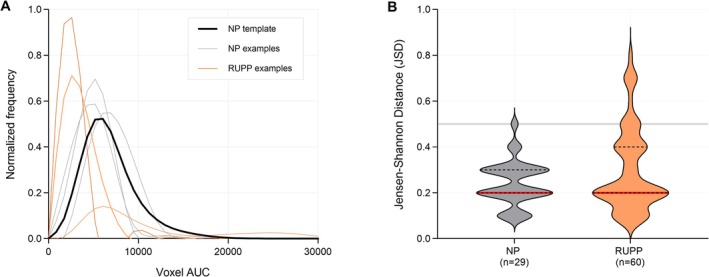
Voxel‐wise histogram analysis of placental perfusion. (A) Normalized voxel‐wise histograms of AUC values showing the NP template (black), representative NP examples (gray), and representative RUPP examples selected from placentas that deviated from the NP template (orange). (B) Violin plots of Jensen–Shannon distance (JSD) relative to the NP template. Median (red) and interquartile range (black) are indicated; the gray horizontal line marks the threshold (mean + 3 SD of NP).

In contrast, histogram‐based analysis revealed marked variability in JSD within the RUPP group. Using a threshold defined as the NP mean + 3 SD, NP placentas clustered clearly below the threshold, whereas JSD values in the RUPP group showed marked intralitter variability, with some resembling NP and others deviating substantially (Figure 4). Overall, 20% of RUPP placentas (12 of 60 analyzed) exceeded the threshold. Two NP outliers exceeded this threshold and were excluded, as they corresponded to a single‐fetus pregnancy and a dam reassigned from the RUPP group due to intraoperative bleeding.

## 4 Discussion

This first application of longitudinal 3D DCE‐MRI in the RUPP model demonstrated acute perfusion deficits after RUPP that resolved rapidly with advancing gestation, consistent with the adaptive placental responses to reduced uterine blood flow.

### 4.1 Maternal and Fetal Outcomes

The observed variability in maternal weight gain and resorption rates highlights the heterogeneous impact of reduced uterine perfusion, consistent with previous reports [13]. Despite a clear early perfusion impairment in all RUPP animals, some RUPP dams maintained normal outcomes, underscoring that the absence of resorptions does not exclude placental ischemia.

Morphometric measures of placental and fetal weights were not different between groups, consistent with earlier work showing variable effects of RUPP on gross morphometry [18, 19]. However, the higher brain‐to‐liver weight ratio observed at GD18 in RUPP fetuses suggests a compensatory brain‐sparing response. Fetal brain sparing is a hallmark of placental insufficiency that has been described in both clinical and experimental settings [20, 21]. This selective redistribution of growth supports the notion that, even in the absence of overt differences in fetal or placental mass, subtle adaptive responses can be detected in the RUPP model.

### 4.2 Placental Perfusion Dynamics

The acute changes in perfusion parameters at GD14 indicate a coherent physiological response to RUPP. Delayed TTP, reduced ME, and reduced WO% reflect reduced inflow and impaired clearance of contrast, consistent with restricted uteroplacental conductance. At the same time, the increase in iAUC despite unchanged total AUC suggests slow vascular transit and prolonged contrast retention, rather than a reduction in overall perfusion volume.

By GD15, partial recovery was evident, with only TTP and WO% remaining abnormal and iAUC trending higher. The normalization of all parameters by GD16–GD18 suggests dynamic adaptation and recovery of perfusion. Together, these semiquantitative measures highlight that early placental ischemia induced by RUPP is transient and recovery involves restoration of flow dynamics without sustained loss of total perfusion capacity. This adaptive response is consistent with laser Doppler findings of reduced uterine blood flow immediately after surgery with partial recovery within 4 days [22]. These findings extend our previous work, where no perfusion differences were observed at GD19 [12], by demonstrating the transient nature of perfusion impairment and subsequent adaptation. The recovery observed here should be interpreted in the context that short‐duration DCE acquisitions primarily reflect maternal perfusion as fetal uptake of gadolinium contrast in murine has been reported only after ≥ 26 min postinjection [23, 24]. Thus, although our results indicate restoration of maternal blood supply, they do not exclude persistent fetal‐side compromise. This interpretation is consistent with our earlier T2* MRI study, which revealed reduced placental oxygenation despite unaltered perfusion, suggesting that maternal–fetal exchange rather than maternal inflow may remain impaired [12].

#### 4.2.1 Semiquantitative DCE‐MRI: Strengths and Limitations

Semiquantitative DCE‐MRI analysis provides a physiologically relevant approach to measure perfusion independently of invasive or model‐based pharmacokinetic assumption [25]. Parameters like TTP, AUC, ME, and WO% reflect perfusion dynamics and efficiency but do not provide absolute physiological values. In this study, semiquantitative parameters were sufficient to capture acute alterations in perfusion after RUPP surgery and to follow their temporal evolution. This approach allowed relative comparisons between groups and across gestational stages, although it should be recognized that it cannot disentangle contributions of blood flow, blood volume, and permeability in the way quantitative modeling might.

Compared with fully quantitative analyses, which require advanced AIF modeling and can be challenging to apply in small animals with rapid circulation, semiquantitative analysis offers a practical compromise. Although it lacks absolute specificity, it enables robust relative assessment and longitudinal monitoring, which was the primary objective of this study.

### 4.3 Voxel‐Wise Histogram Analysis of Intralitter Variability

Placenta‐level mean and median voxel‐wise AUC did not demonstrate a consistent group‐level shift at GD15, indicating that voxel‐wise averaging alone does not separate NP from RUPP at this time point. Voxel‐wise histogram analysis was therefore used as an exploratory approach to capture distributional differences and within‐litter variability that may not be reflected by average AUC values.

Two NP placentas initially exceeded the JSD threshold and were excluded as both were associated with specific biological or procedural factors. Their exclusion ensured a robust NP reference template, while also highlighting the sensitivity of voxel‐wise analysis to detect atypical perfusion patterns.

Within RUPP litters, many placentas maintained NP‐like perfusion profiles, whereas only 20% showed clear deviations. This heterogeneity likely reflects differences in vascular remodeling or positional effects along the uterine horn. Although exploratory, this voxel‐wise histogram may therefore help distinguish “resilient” from “compromised” placentas, underscoring its value as a complement to conventional ROI methods for characterizing within‐litter variation.

### 4.4 Implications for Timing of Assessment and Intervention

The longitudinal design of this study enabled a comprehensive evaluation of placental perfusion using repeated 3D DCE‐MRI in the RUPP rat model. The impact of reduced uterine perfusion was strongly time dependent. The transient impairment and subsequent recovery underscore the importance of gestational timing when assessing placental function. The early window at GD14–GD15 appears most relevant for interventions targeting the acute perfusion deficit induced by RUPP. However, we do not exclude benefit from later interventions aimed at secondary injury cascades including the reduced placental oxygenation at GD19 in this model [12] and the higher brain‐to‐liver ratio we observed at GD18. Thus, although early measures may help preserve fetuses otherwise at risk of resorption, later therapies may still modify downstream pathology and growth trajectories. Finally, because RUPP imposes an abrupt reduction in uterine blood flow whereas human placental insufficiency typically evolves more gradually, these timing considerations should be factored into the design, interpretation, and translation of therapeutic studies using the RUPP model.

### 4.5 Limitations

This study has several limitations that should be considered when interpreting the findings. First, the follow‐up period was restricted to 3 days due to animal welfare considerations, which limited our ability to assess longer term adaptations (e.g., at GD19–GD21). In addition, repeated anesthesia across sessions may have introduced confounding effects, although no overt adverse outcomes were observed. A further methodological consideration is that, to minimize the impact of multiple contrast injections within the same day, group comparisons at GD14 were made between RUPP and NP animals rather than within the same animal presurgery and postsurgery. This approach constrained within‐animal baseline assessments but still revealed a clear reduction in perfusion after surgery. Additionally, the voxel‐wise analysis required highly time‐consuming manual segmentation, limiting the number of placentas used for this analysis. This may be facilitated in future studies by the use of AI‐based segmentation algorithms. Finally, the short acquisition window means that the results primarily reflect materno–placental perfusion and provide limited insight into the fetal circulation. Pairing DCE‐MRI with oxygenation measures from T2* MRI and photoacoustic imaging may provide a more complete picture of both maternal and fetal contributions to placental function.

## 5 Conclusion

This study demonstrates that longitudinal 3D DCE‐MRI can detect acute placental perfusion impairment immediately after RUPP surgery, followed by recovery at later gestational stages. These findings provide new insights into the temporal dynamics of placental adaptation to reduced uterine blood flow and identify an early window for potential therapeutic intervention.

## Author Contributions

**Fatimah M. Al Darwish:** writing – original draft, writing – review and editing, visualization, methodology, investigation, formal analysis, data curation. **Caren M. van Kammen:** writing – review and editing, methodology. **Fieke Terstappen:** writing – review and editing, supervision, project administration, methodology, conceptualization. **Lindy K. Alles:** writing – review and editing, investigation. **Raymond M. Schiffelers:** writing – review and editing, funding acquisition. **A. Titia Lely:** writing – review and editing, supervision, funding acquisition, conceptualization. **Gustav J. Strijkers:** writing – review and editing, supervision, resources, funding acquisition, conceptualization. **Bram F. Coolen:** writing – original draft, writing – review and editing, project administration, methodology, investigation, conceptualization.

## Funding

This work was supported by ZonMw, the Netherlands Organization for Health Research and Development (Grant No. TRIPLET NWO 24434).

## Conflicts of Interest

The authors declare no conflicts of interest.

## Supporting information


**Video S1:** 3D DCE‐MRI of representative placentas from NP and RUPP dams at GD14 and GD16. At GD14, RUPP placentas show delayed and attenuated enhancement relative to NP; at GD16, enhancement is similar to NP, indicating recovery. For display, a single slice from the 3D volume is shown capturing most placentas.

## Data Availability

The data that support the findings of this study are available from the corresponding author upon reasonable request.
