# Supplementary figures and images for: Longitudinal 3D‐DCE MRI Assessment of Placental Perfusion Under Reduced Uterine Perfusion Pressure in Pregnant Rats
Source: NMR Biomed. 2026 Feb 22;39(4):e70251. doi: 10.1002/nbm.70251 (PMC12926275; doi:10.1002/nbm.70251)

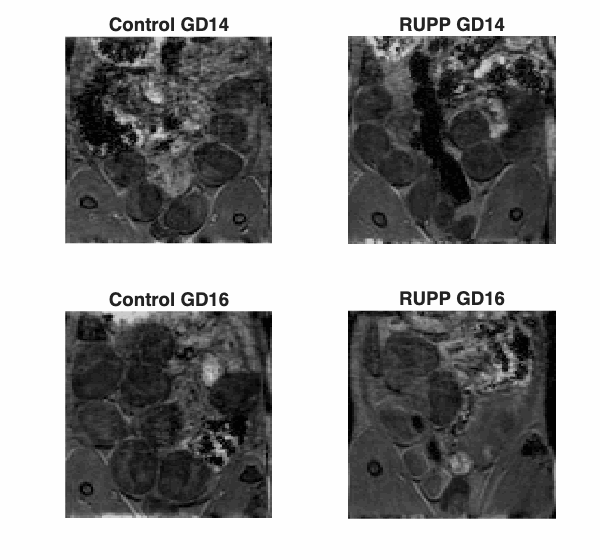

Supplement: Supplementary file 1 — Video S1: 3D DCE‐MRI of representative placentas from NP and RUPP dams at GD14 and GD16. At GD14, RUPP placentas show delayed and attenuated enhancement relative to NP; at GD16, enhancement is similar to NP, indicating recovery. For display, a single slice from the 3D volume is shown capturing most placentas. [file NBM-39-e70251-s001.gif]
